# Effect of In-Utero Antibiotic Exposure on Childhood Outcomes: Methods and Baseline Data of the Fetal Antibiotic EXposure (FAX) Cohort Study

**DOI:** 10.2196/12065

**Published:** 2019-07-30

**Authors:** Corinna Koebnick, Sara Y Tartof, Margo A Sidell, Emily Rozema, Joanie Chung, Vicki Y Chiu, Zackary W Taylor, Anny H Xiang, Darios Getahun

**Affiliations:** 1 Kaiser Permanente Southern California Research and Evaluation Pasadena, CA United States; 2 Kaiser Permanente Department of Research and Evalutaion Pasadena, CA United States; 3 Kaiser Permanente Pediatrics Los Angeles, CA United States

**Keywords:** pregnancy, antibiotic, pediatric obesity, asthma, Outcome Assessment (Health Care)

## Abstract

**Background:**

The widespread use of antepartum and intrapartum antibiotics has raised concerns about the possible disruption of the child’s gut microbiota and effects on the maturation from the infant to the adult microbiome. The Fetal Antibiotic EXposure (FAX) study provides a cohort to examine the association between *in-utero* exposure to antibiotics and adverse childhood outcomes including body weight, atopic diseases, and autism spectrum disorders and to investigate the role of other potential factors mitigating or moderating the risk for adverse outcomes.

**Objective:**

The aim of this paper was to describe the methods, cohort characteristics, and retention of infants included in the study cohort.

**Methods:**

For this retrospective cohort study, we included children born in Kaiser Permanente Southern California (KPSC) hospitals between January 1, 2007, and December 31, 2015, within 22 to 44 completed weeks of gestation with KPSC insurance coverage during the first year of life. Follow-up data collection was performed through electronic medical records.

**Results:**

The study cohort was comprised 223,431 children of which 65.7% (146,720/223,431) were exposed to antibiotics *in-utero*: 19.0% (42,511/223,431) were exposed during the antepartum period, 30.0% (66,896/223,431) during the intrapartum period, and 16.7% (37,313/223,431) exposed during both the antepartum and intrapartum periods.

During their first year of life, children had a median of 5 weight and height measurements; the frequency of weight and height measurements declined to a median of 3 in their second year of life and 2 for 3 to 5 years of age. The 5-year retention of children in the health plan was over 80% with the highest retention for Hispanic children.

**Conclusions:**

This cohort of children will provide a unique opportunity to address key questions regarding the long-term sequelae of *in-utero* exposure to antibiotics using real-world data. The high retention and multiple medical visits over time allow us to model the trajectories of body mass index over time.

**International Registered Report Identifier (IRRID):**

DERR1-10.2196/12065

## Introduction

Antibiotics are frequently used to prevent and treat infections during pregnancy [1-3]. Antepartum, respiratory, and urinary tract infections as well as pelvic inflammatory disorder are common reasons for antibiotic use [3]. During the intrapartum period, the use of antibiotics is frequent in high-risk women to prevent vertical transmission and early-onset group B streptococcal (GBS) diseases in neonates with an estimated prevalence of GBS colonization in 10% to 30% of pregnant women [4-6]. Moreover, surgical antibiotic prophylaxis is administered to about 19% of women before cesarean incision [7-10]. In Canada, the United States, and Europe, about 40% of pregnant women received antibiotics according to studies using data between 1998 and 2010 [2,3,11]. Although antibiotics are indicated for obstetrical and nonobstetrical conditions in pregnant women, little is known about medium- and long-term health effects in the infant as the consequence of *in-utero* exposure to antibiotics.

The widespread use of antibiotics during antepartum and intrapartum periods has raised concerns about alteration of the microbiota of the maternal birth canal before birth and interference with the early microbial transfer from the mother to the fetus during pregnancy, delivery, and lactation. This may lead to disruption of the child’s gut microbiota [12,13]. The bacterial microbiome in infants undergoes changes until it develops into a more stable adult-like microbiome [14]. Early life disruptions in microbial colonization and maturation may have downstream consequences on the metabolism and health of a child [15,16]. However, it is unclear as to how the exposure to antibiotics *in-utero* affects the *maturation* from the infant to the adult microbiome and, in consequence, how this may affect the health of a child.

**Figure 1:**
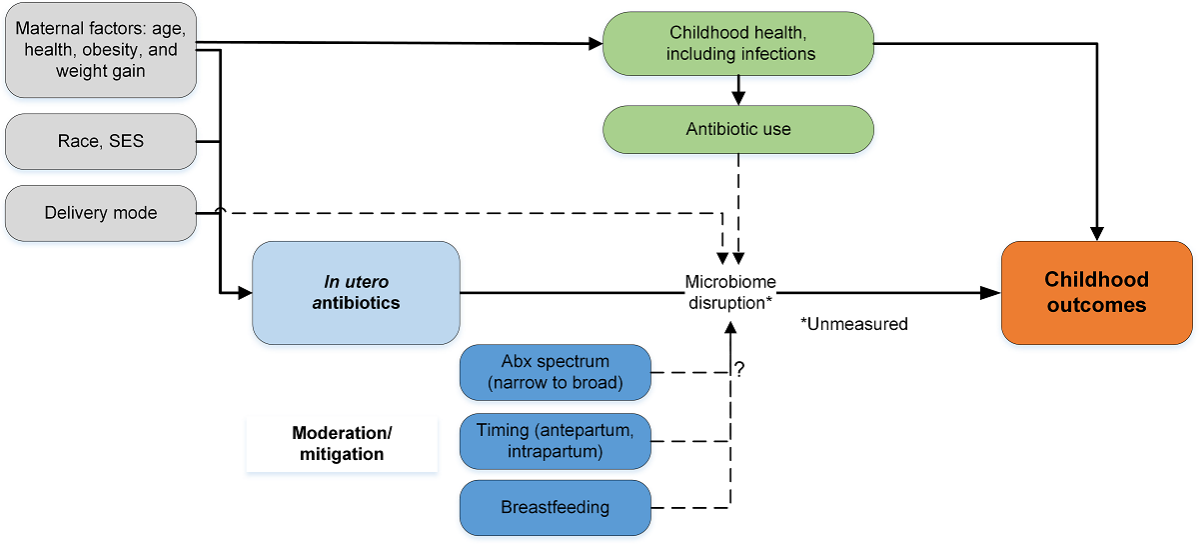
Conceptual model of the Fetal Antibiotic EXposure (FAX) study.

The aims of the Fetal Antibiotic EXposure (FAX) study are to examine the association between *in-utero* exposure to antibiotics and adverse childhood outcomes including body weight, atopic diseases, and autism spectrum disorders (Figure 1) and to investigate the role of other potential factors mitigating or moderating the risk for adverse outcomes. Here, we describe the methods, cohort characteristics, and retention of infants included in the study cohort.

## Methods

### Setting and Study Design

For this retrospective cohort study, we used longitudinal electronic medical record data from Kaiser Permanente Southern California (KPSC), the largest integrated health care system in Southern California. KPSC provides comprehensive health services to over 4 million health plan members. The membership represents approximately 17.0% of the population in the coverage area and has similar sociodemographic characteristics such as neighborhood-level education and income [17]. Medical services are provided almost solely in KPSC-owned hospitals and medical offices. Labor, delivery, and pharmacy records contain detailed data on drug dispenses and administration. About 42,000 babies were delivered in KPSC hospitals in 2017. All children are linked with their biological mothers using unique identifiers. For the assessment of study outcomes, infants will be followed longitudinally using information from the electronic medical records. The study protocol was reviewed and approved by KPSC’s Institutional Review Board.

### Study Population

**Figure 2:**
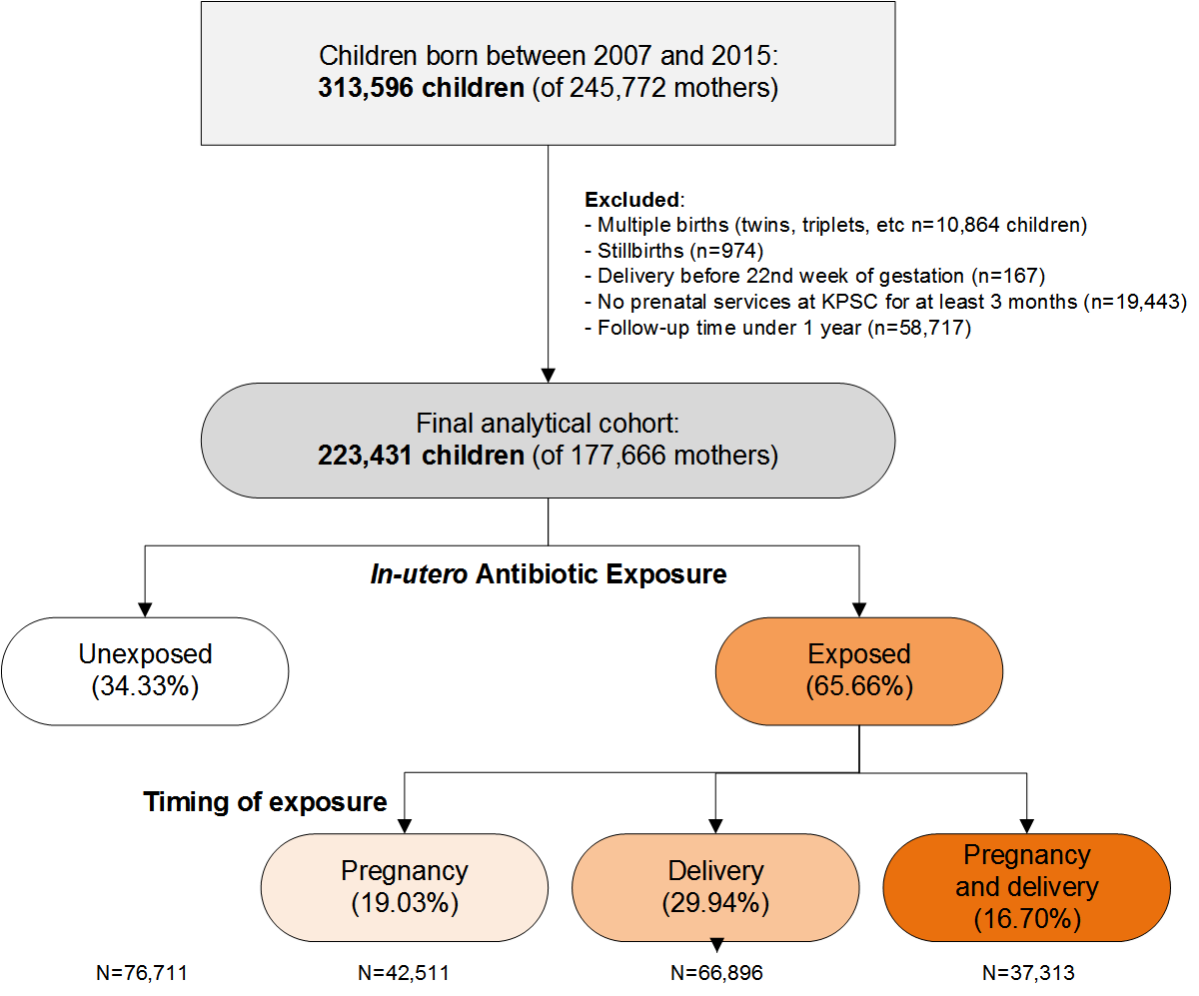
Flow chart of infants enrolled in the FAX study by in-utero exposure to antibiotics. KPSC, Kaiser Permanente Southern California.

We included children born in KPSC hospitals between January 1, 2007, and December 31, 2015, within 22 to 44 completed weeks of gestation with KPSC insurance coverage during the first year of their life. We excluded infants from multiple births, infants with less than 2 medical encounters with documented vital signs including weight and height measurements during follow-up, and infants whose mothers did not have KPSC health insurance coverage for at least 3 months before delivery to be able to assess *in-utero* antibiotic exposure antepartum. After applying all inclusion and exclusion criteria, the cohort size was 223,431 children (71% of all children born in KPSC hospitals; Figure 2).

### Exposure Ascertainment

Primary exposure of interest is the *in-utero* exposure to antibiotics during pregnancy and includes oral, intramuscular, and intravenous administration. We divided the timing of antibiotic exposure into (1) time between conception and onset of labor (or admission for delivery) and (2) intrapartum, defined as the time between maternal admission for labor and delivery of the infant. For cases with longer inpatient stays before delivery, the intrapartum period is defined as 48 hours before delivery order to distinguish false labor from true labor.

*Ex-utero* exposure to antibiotics during childhood (including the neonatal period) and as indirect exposure to maternal antibiotic use during lactation will be extracted to control for independent effect of *ex-utero* exposure on the outcome of interest.

### Outcome Ascertainment

#### Childhood Body Weight and Obesity

At KPSC, body weight and height are routinely measured by trained staff on calibrated scales at almost every medical office visit. In pediatrics and family practice, staff must complete a Web-based training session and successfully pass a certification process that includes knowledge of preparing patients for measuring weight and height and their competency is assessed. Data on weight and height will be extracted from all medical encounters. Biologically implausible values for weight and height data will be excluded [18,19]. Body mass index (BMI; kg/m^2^) will be used as untransformed BMI instead of sex-specific BMI-for-age percentiles developed by the Centers for Disease Control and Prevention [20] because estimates are more interpretable [21-23].

#### Other Outcomes

Childhood asthma and atopic disease will be extracted from electronic medical records. Pediatric asthma is defined as (1) physician-diagnosed asthma (*International Classification of Diseases*, 9th revision, Clinical Modification codes ICD-9-CM 493.xx, ICD-10 J45.xx) and at least 2 prescriptions specific to asthma medication (beta-agonists or asthma controller medications or their combination) in year 1 and 2 after diagnosis or (2) physician-diagnosed wheezing (ICD-9-CM 786.07, ICD-10 R06.2) and at least 2 prescriptions specific to asthma medication (beta-agonists or asthma controller medications or their combination) in year 1 and 2 after diagnosis. Atopic disease is defined as physician-diagnosed allergic rhinitis (ICD-9-CM 477.x, ICD-10 J30.9), asthma, or atopic dermatitis (ICD-9-CM 691.8, ICD-10 L20.xx). Other outcomes may be added over time.

### Demographic and Other Factors

Race and ethnicity information was obtained from health plan electronic medical records and administrative and birth records before 2011. As part of the implementation of meaningful use requirements for electronic medical records [24], self-reported race and ethnicity was collected systematically from members starting 2011. Typically, new and current members were asked to complete a self-report form that included separate questions for both their race and ethnicity. These forms were included in both membership applications and at clinical outpatient visits. The choices for race and ethnicity recorded were standardized across health care systems and followed national recommendations for mutually exclusive race categories [25,26]. Regardless of the race category endorsed, patients self-reporting Hispanic ethnicity were considered Hispanic according to recommendations from a national survey of Hispanics living in the United States that Hispanic people considered themselves a race of people and not an ethnicity [27]. If a patient’s records contained 2 or more race categories (rather than a single category of *mixed race*), they were assigned the least prevalent race category in the US population. For example, if a patient indicated that they were both Native Hawaiian and Pacific Islander and non-Hispanic black, they were categorized as Native Hawaiian or Pacific Islander in our analyses. This was done to maximize our ability to understand differences in diagnoses and treatment for the least represented racial and ethnic minority patients. This is a convention used across many health care organizations using a standardized virtual data warehouse [28]. If race and ethnicity were missing, the available language preferences were used to impute race and ethnicity for Hispanics and Asians. We categorized race and ethnicity as non-Hispanic white (white), Hispanic (regardless of race), non-Hispanic black (black), Asian and Pacific Islander (API), and other or unknown race or ethnicity.

Neighborhood-level (based on census tract) education, neighborhood-level household income, and the neighborhood proportion of individuals below poverty line were used to indicate socioeconomic status. These population-level indicators were estimated by geocoding cohort members’ addresses to 2010 US census block data [29]. Maternal education (individual-level data) is available from birth records. We also included insurance coverage through government health care assistance programs such as Medicaid as an additional proxy for socioeconomic status.

Gestational age is based on the clinical estimate of gestational age as recorded in the maternal electronic medical records [30]. Fetal growth is defined on the basis of the 2000 to 2015 race - and sex-specific nomogram (internal standard) and classified as small-for-gestational age when birth weight is less than the tenth or fifth percentile for gestational age [31,32].

Information on maternal medical and obstetrical history of the index pregnancy includes BMI, gestational weight gain, pregestational and gestational diabetes, and chronic and gestational hypertension. Maternal asthma is defined as physician-diagnosed asthma (ICD-9-CM 493.xx and ICD-10 J45.xx) before birth.

Breastfeeding was documented in progress notes (unstructured text format) between 2007 and 2010. To extract this information, we developed a natural language processing algorithm [33]. The overall ability of the natural language processing algorithm in accurately extracting breastfeeding status was assessed using manual chart review of a random sample of medical record notes from 500 children. Sensitivity, specificity, and positive and negative predictive values for breastfeeding for >6 months detected by natural language processing were 89%, 89%, and 83% and 93%, respectively [33]. After 2010, breastfeeding information was assessed from surveys administered during or before well-child visit surveys at well-baby health care visits at birth to 4 weeks, 2 months, 4 months, 6 months, 9 months, and 12 months of life [33]. Surveys are completed by the parent guardian; collection modes include patient portal, paper (with later data entry by medical support staff), and tablet computers in the waiting room. All survey answers are structured data fields embedded in electronic medical records [34].

### Statistical Analysis

For analyses presented here, we compared clinical and demographic characteristics between *in-utero* antibiotic exposure groups using descriptive statistical methods including Pearson chi-square test or Fisher exact test (if data are sparse) for categorical variables and using analysis of variance or the Kruskal-Wallis test for continuous variables depending on the distribution. Maternal BMI before pregnancy was categorized as <18.5, 18.5 to 22.4, 22.5 to 24.9, 25.0 to 29.9, 30.0 to 34.9, 35.0 to 39.9, and ≥40 kg/m^2^. The start of prenatal care was divided into a first visit <3, 4 to 6, >6 months of gestation and no care visits. Retention in the health plan was calculated as the proportion of children with health care coverage at any time within a certain year. All analyses were performed using SAS Enterprise Guide 5.1 (SAS Institute).

## Results

The study cohort was comprised 114,464 male and 108,967 female infants born in KPSC hospitals between 2007 and 2015 to 177,666 mothers. About 67,050 out of 223,431 infants (30.00%) were born via cesarean section, whereas 17,191 out of 223,431 (7.69%) were born preterm. Approximately half of the infants were non-Hispanic whites (Multimedia Appendix 1). Almost 1 out of 10 infants (n=20,071, 8.98%) had insurance coverage through government health care assistance programs. Most mothers (n=199,099, 89.11%) initiated their prenatal care at KPSC in their first trimester of pregnancy; about 22.52% of mothers (n=50,312) were obese with 9.59% of mothers (n=21,436) having a BMI ≥35 kg/m^2^.

The *in-utero* exposure to antibiotics was frequent with 42,511 out of 223,431 (19.03%) of infants exposed during the antepartum period, 66,896 out of 223,431 (29.94%) during the intrapartum period, and 3731 out of 223,431 (16.69%) exposed during both the antepartum and intrapartum periods. About one-third of infants (76711 out of 223431, 34.33%) were not exposed to antibiotics *in-utero* (Multimedia Appendix 1). Compared with non-Hispanic white infants, black infants were more likely to be exposed to antibiotics *in-utero* overall; Hispanic infants were more likely to be exposed to antibiotics during pregnancy, and API infants were more likely to be exposed to antibiotics during the intrapartum period. Infants born preterm, with insurance coverage through government health care assistance programs, born to older mothers, born to mothers with a higher BMI, and born to mothers who smoked during pregnancy were more likely to be exposed to antibiotics *in-utero*.

The 5-year retention of infants in the health plan was over 80% (Table 1) and it has been increasing steadily since 2007. Retention was the lowest for infants with unknown race or ethnicity and highest for Hispanic infants. The retention of infants did not differ between infants with and without insurance coverage through government health care assistance programs.

**Figure 3:**
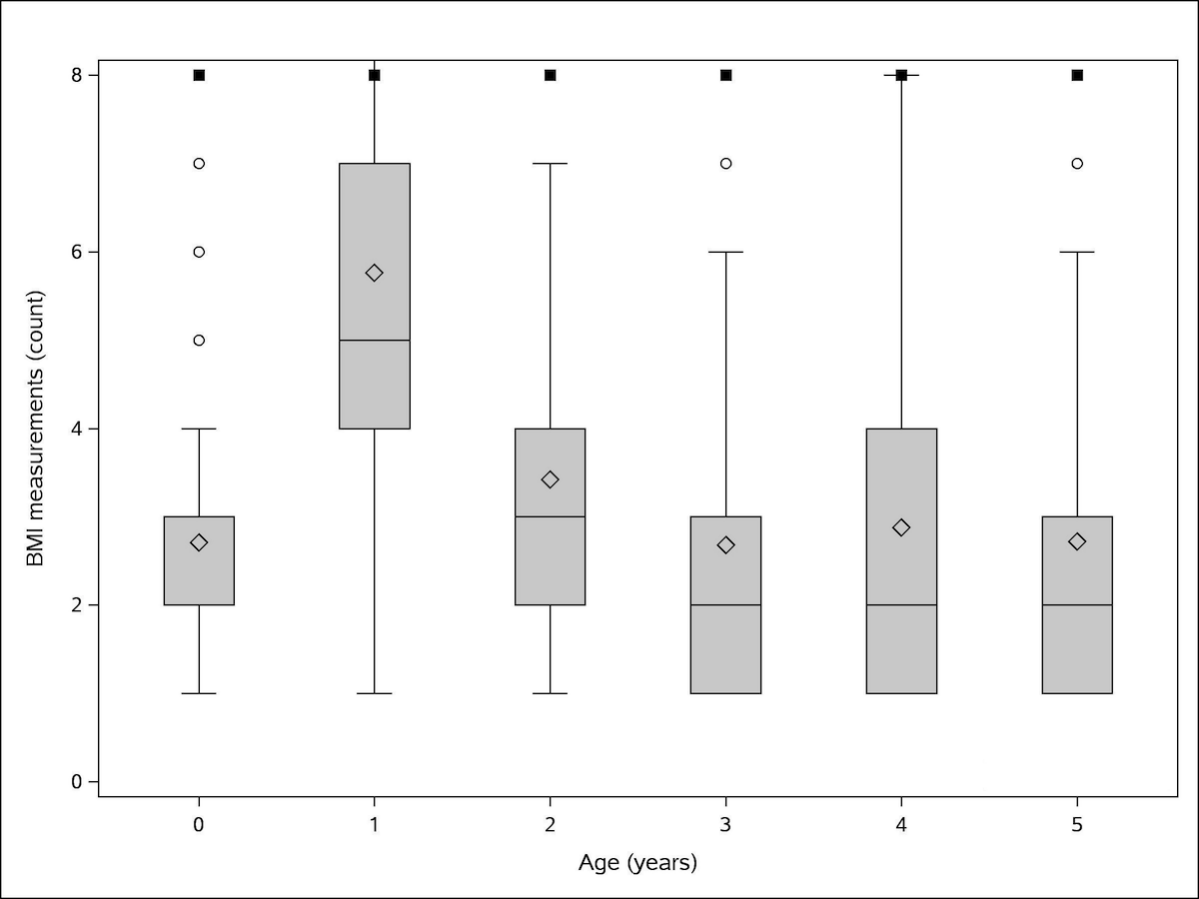
Box-and-whisker plot of frequency of body mass index measures per child by age (Age 0 includes the neonatal period defined as the first 28 days of life. Plot boxes reflect the interquartile range (IQR; 25th and 75th percentile) as box limits, median (blue line), and mean (blue diamond). Whiskers reflect the minimum and maximum within 1.5 x IQR. Outliers (black circles) were cut at a maximum of 8 measurements). BMI: body mass index.

During the neonatal period, infants had a median of 3 BMI measurements recorded and during their first year of life, a median of 5 BMI measurements (Figure 3). The number of recorded BMI measurements per child and year then slowly declined to 2 in the fifth year of life. The availability of BMI measures was consistent across all birth cohorts from 2007 to 2015 (data not shown).

**Table 1:** Retention of infants by birth cohort, race and ethnicity, and government health care assistance.

Variable		Retention (years), n (%)			
		Baseline	1	3	5
**Birth cohort**					
	2007	22,397 (10.02)	22,375 (99.90)	18,769 (83.80)	17,402 (77.70)
	2008	23,242 (10.40)	23,219 (99.90)	19,849 (85.40)	18,245 (78.50)
	2009	23,066 (10.32)	23,066 (100.00)	19,814 (85.90)	18,061 (78.30)
	2010	23,294 (10.43)	23,294 (100.00)	19,916 (85.50)	18,542 (79.60)
	2011	24,365 (10.90)	24,341 (99.90)	20,759 (85.20)	19,638 (80.60)
	2012	25,621 (11.47)	25,595 (99.90)	22,085 (86.20)	20,753 (81.00)
	2013	25,632 (11.47)	25,632 (100.00)	22,505 (87.80)	—^a^
	2014	26,985 (12.08)	26,985 (100.00)	23,774 (88.10)	—
	2015	28,829 (12.90)	28,800 (99.90)	—	—
**Race and ethnicity**					
	White	63,085 (28.23)	63,085 (100.00)	52,991 (84.00)	49,017 (77.70)
	Hispanic	105,699 (47.31)	105,593 (99.90)	92,698 (87.70)	88,153 (83.40)
	Black	18,934 (847)	18,915 (99.90)	16,529 (87.30)	15,621 (82.50)
	Asian and Pacific Islander	27,937 (12.50)	27,937 (100.00)	24,389 (87.30)	23,048 (82.50)
	Others or Unknown	7776 (3.48)	7768 (99.90)	6182 (79.50)	5669 (72.90)
**Government health care assistance**					
	No	203,360 (91.02)	203,157 (99.90)	175,703 (86.40)	165,128 (81.20)
	Yes	20,071 (8.98)	20,071 (100.00)	17,241 (85.90)	16,418 (81.80)

^a^Not applicable.

## Discussion

In total, 2 out of 3 infants included in the FAX study were exposed to antibiotics *in-utero*; the exposure primarily occurred during the intrapartum period. The use of antibiotics during pregnancy is a risk-versus-benefit decision because untreated infections are associated with significant risk for the unborn child [1,35,36]. Owing to the widespread use of antibiotics, understanding the short- and long-term risks of *in-utero* exposure to antibiotics is an important public health issue. Considering the high proportion of infants exposed, even small individual-level health risks from antibiotic exposure could result in significant population-level effects. Possible adverse childhood outcomes include microbiome disruption [37], obesity [38,39], and infections [40,41]. Future interventions can be targeted to attenuate the risk [42-47].

To address current gaps in the knowledge about the health consequences of *in-utero* antibiotic exposure, we created a large cohort study of infants with racial, ethnic and socioeconomic diversity. The large sample size is particularly useful to support the study of rare outcomes and smaller population segments defined by race and socioeconomic status. Available clinical information is robust and reflects *real-world* information that clinicians and health plans use to document health care rather than research-quality data collected at prespecified study intervals [48].

Weight and height were frequently measured and recorded in the electronic medical records for calculation of BMI [19]. The frequency of BMI measurements per child together with the large sample size will allow us to model nonlinear trajectories of BMI or identify distinct BMI trajectories. We will be able to model these trajectories for the overall cohort and for the strata of children defined by the *in-utero* exposure to antibiotics ante- and intrapartum.

Several challenges for studies using data from the FAX cohort are noted. The use of routinely measured clinical weight and height may increase variation, which may bias potential risks toward the null. However, this bias will be compensated by the large sample size. Another source of variation is the variation in medical practice. In an ideal world, acute medical conditions such as infections are treated according to published recommendations. This may not always be the case in clinical practice. On the contrary, practice variation and the varying probability to be treated can be addressed in analyses by using appropriate statistical methods. In fact, practice variation can add knowledge about real-world settings, which can be readily translated into clinical practice.

Missing values may be frequent in routinely collected electronic medical record data and may not always be missing at random but they reflect real-world settings. The data compiled for the proposed study were collected for routine clinical care and not specifically for research purposes. However, most information such as race, ethnicity, and breastfeeding were collected for almost all patients [33,49]. To address any possible bias because of missing baseline or follow-up outcomes or covariates, we will examine baseline characteristics to determine if there are any systematic differences between the groups with and without missing data. If differences are found, appropriate estimation methods can be used to account for biases because of missing data, such as multiple imputations. Although addressing these challenges poses difficulty in the proposed research, the use of real-world clinical data also represents a key strength of the study.

Another potential challenge is the possibility of attrition because of patients leaving the health plan. KPSC is an open health care plan and members can enroll and disenroll. However, retention of members in the health plan, especially in the targeted age group, is high. To better understand the effects of attrition on the cohort characteristics, we will compare the characteristics (eg, demographics and BMI) of those who remain enrolled in KPSC and those who discontinued.

The current cohort of children will provide a unique opportunity to address key questions regarding the long-term sequelae of *in-utero* exposure to antibiotics in real-world data. The cohort shows a high retention rate and provides data from multiple medical visits over time, allowing us to model the trajectories of BMI over time.

## Appendix

Multimedia Appendix 1 Characteristics of infants born 2007-2015 and enrolled in the Fetal Antibiotic EXposure Study.

## Abbreviations

API: Asian and Pacific Islander
BMI: body mass index
FAX: Fetal Antibiotic EXposure
GBS: group B streptococcal
KPSC: Kaiser Permanente Southern California
