# Supplementary material for: Effect of In-Utero Antibiotic Exposure on Childhood Outcomes: Methods and Baseline Data of the Fetal Antibiotic EXposure (FAX) Cohort Study
Source: JMIR Res Protoc. 2019 Jul 30;8(7):e12065. doi: 10.2196/12065 (PMC6691671; doi:10.2196/12065)
Supplement: Multimedia Appendix 1 [file resprot_v8i7e12065_app1.pdf]

Multimedia Appendix:

Characteristics of infants born 2007-2015 and enrolled in the FAX study.

	**Total**	***In-utero* exposure to antibiotics**			
Variable	**Infant cohort**	**None**	**Pregnancy[a]**	**Intrapartum**	**Pregnancy[a]+ Intrapartum**
n (%)	223431	76711	42511	66896	37313
**Sex**, male	114464 (51.23)	38325 (49.96)	21513 (50.61)	35101 (52.47)	19525 (52.33)
**Race and Ethnicity**					
White	63085 (28.23)	22866 (29.81)	11261 (26.49)	19560 (29.24)	9398 (25.19)
Hispanic	105699 (47.31)	36238 (47.24)	22047 (51.86)	29366 (43.90)	18048 (48.37)
Black	18934 (8.47)	4675 (6.09)	4138 (9.73)	5417 (8.10)	4704 (12.61)
API	27937 (12.50)	10153 (13.24)	3781 (8.89)	10010 (14.96)	3993 (10.70)
Others and Unknown	7776 (3.48)	2779 (3.62)	1284 (3.02)	2543 (3.80)	1170 (3.13)
**Maternal education**					
<High school	10969 (4.91)	4020 (5.24)	2588 (6.09)	2678 (4.00)	1683 (4.51)
High school	55668 (24.92)	18879 (24.61)	11979 (28.18)	15211 (22.74)	9607 (25.75)
Some college	67772 (30.30)	21750 (28.35)	14067 (33.09)	19530 (29.19)	12425 (33.30)
Bachelor degree	54001 (24.17)	19496 (25.41)	8485 (19.96)	17825 (26.65)	8195 (21.96)
Graduate or higher	32036 (14.34)	11537 (15.04)	4867 (11.45)	10736 (16.05)	4896 (13.12)
Unknown	2985 (1.34)	1037 (1.35)	525 (1.23)	916 (1.37)	507 (1.36)
**Neighborhood income**					
< $15,000	22657 (10.14)	7646 (9.97)	4435 (10.43)	6683 (9.99)	3893 (10.43)
$15,000 to $34,999	38582 (17.27)	12957 (16.89)	7369 (17.33)	11459 (17.13)	6798 (18.22)
$35,000 to $49,999	31015 (13.88)	10611 (13.83)	6030 (14.18)	9158 (13.69)	5217 (13.98)
$50,000 to $74,999	43042 (19.26)	14769 (19.25)	8273 (19.46)	12825 (19.17)	7175 (19.23)
$75,000 to $99,999	30944 (13.85)	10674 (13.91)	5842 (13.74)	9321 (13.93)	5107 (13.69)
$100,000 to $149,999	33105 (14.82)	11516 (15.01)	6109 (14.37)	10079 (15.07)	5400 (14.47)
≥$150,000	20780 (9.30)	7280 (9.49)	3629 (8.54)	6499 (9.72)	3373 (9.04)
**Government health care assistance**	20071 (8.98)	5842 (7.62)	4922 (11.58)	4849 (7.25)	4458 (11.95)
**Preterm birth**#	17191 (7.69)	2201 (2.86)	2117 (4.98)	7136 (10.67)	5737 (15.38)
**Delivery mode**					
Vaginal	156381 (70.00)	76711 (100.00)	36982 (86.99)	24973 (37.33)	17715 (47.48)
Cesarean section	67050 (30.00)	0 (0.00)	5529 (13.01)	41923 (62.67)	19598 (52.52)
**Small-for-gestational age**	22026 (9.86)	7712 (10.05)	4287 (10.08)	6486 (9.70)	3541 (9.49)
**Maternal age**, years					

<20	7900 (3.54)	2745 (3.58)	2299 (5.41)	1477 (2.21)	1379 (3.70)
20-29	91292 (40.86)	32464 (42.32)	19445 (45.74)	24301 (36.33)	15082 (40.42)
30-34	73787 (33.02)	26200 (34.15)	12699 (29.87)	23245 (34.74)	11643 (31.20)
≥35	50452 (22.58)	15302 (19.95)	8068 (18.98)	17873 (26.72)	9209 (24.68)
**Maternal pre-pregnancy BMI**, kg/m2					
<18.5	4733 (2.12)	1910 (2.49)	960 (2.26)	1167 (1.74)	696 (1.87)
18.5-22.4	46387 (20.76)	17909 (23.35)	8481 (19.95)	13167 (19.68)	6830 (18.30)
22.5-24.9	38861 (17.39)	14047 (18.31)	7132 (16.78)	11464 (17.14)	6218 (16.66)
25.0-29.9	54718 (24.49)	17889 (23.32)	10607 (24.95)	16512 (24.68)	9710 (26.02)
30.0-34.9	28876 (12.92)	7847 (10.23)	5653 (13.30)	9220 (13.78)	6156 (16.50)
35.0 -39.9	13386 (5.99)	3036 (3.96)	2589 (6.09)	4409 (6.59)	3352 (8.98)
≥40	8050 (3.60)	1480 (1.93)	1452 (3.42)	2795 (4.18)	2323 (6.23)
Missing	28420 (12.72)	12593 (16.42)	5637 (13.26)	8162 (12.20)	2028 (5.44)
**Maternal smoking during pregnancy**	24231 (10.84)	7114 (9.27)	5244 (12.34)	6946 (10.38)	4927 (13.20)
**Start of prenatal care**					
≤ 3 months	199099 (89.11)	67659 (88.20)	38289 (90.07)	59506 (88.95)	33645 (90.17)
4-6 months	19922 (8.92)	7306 (9.52)	3435 (8.08)	6086 (9.10)	3095(8.29)
> 6 months	2386 (1.07)	982 (1.28)	360 (0.85)	759 (1.13)	285 (0.76)
No care or missing	2024 (0.91)	764 (1.00)	427 (1.00)	545 (0.82)	288 (0.77)
**Parity**					
1	168219 (75.29)	57128 (74.47)	30932 (72.76)	52279 (78.15)	27880 (74.72)
2	36208 (16.21)	12848 (16.75)	7384 (17.37)	9846 (14.72)	6130 (16.43)
≥3	19004 (8.51)	6735 (8.78)	4195 (9.87)	4771 (7.13)	3303 (8.95)

[a]Pregnancy without intrapartum period

<37 week of gestation
